# Unexplained pancytopenia in acute myeloid leukemia treatment

**DOI:** 10.1002/ccr3.1570

**Published:** 2018-06-19

**Authors:** Daniel Ezekwudo, Oleksandra Lupak, Raju Vaddepally, Bolanle Gbadamosi, Philip Kuriakose

**Affiliations:** ^1^ Department of Hematology and Oncology Henry Ford Hospital Detroit MI USA; ^2^ Department of Hematology and Oncology William Beaumont Hospital Royal Oak MI USA

**Keywords:** acute myeloid leukemia, bone marrow necrosis, chemotherapy sensitivity, hemophagocytic lymphohistiocytosis, induction chemotherapy, pancytopenia

## Abstract

Certain histopathological findings have been described in acute myeloid leukemia (AML) patients during treatment that define the hematologic outcomes. Such entities as bone marrow necrosis and hemophagocytic lymphohistiocytosis have been reported. These often result in severe pancytopenia.

A 61‐year‐old male with severe rheumatoid arthritis, prostate CA (s/p prostatectomy) presented to the emergency department due to abnormal CBC from primary care physician. Initial laboratory revealed WBC 26.3 K/uL with absolute monocytes of 15.52 K/uLl, hemoglobin 7.4 g/dL, and platelet count 142 K/uL. Bone marrow revealed acute myeloid leukemia with monocytic differentiation and dysplasia. FISH revealed no rearrangements in 8q22 (RUNX1T1—ETO), 11q23 (5′MLL, 3′MLL), 15q22 (PML), CBFB break apart for inv(16) or t(16;16), 17q21 (RARA), and 21q22 (RUNX1 ‐ AML1). Over the next day, his WBC increased to 121 K/uL with abnormal liver enzymes. He also complained of blurry vision and mild persisting headache; raising the question of possible CNS infiltration by leukemic cells. Induction therapy utilizing 3 + 7 with Idarubicin and cytarabine was initiated. Three‐five days into treatment, patient developed severe pancytopenia (WBC 2.9, Hgb 6.3, Plt < 10) and neutropenia (0.49 K/uL) (Figure 1A). Peripheral blood smears review showed classic hemophagocytes (depicted in Figure 1B). The sudden cytopenia on day three is unusual for AML regardless of the type, thus raising the question as to whether there are other competing pathologic processes involved.

**Figure 1:**
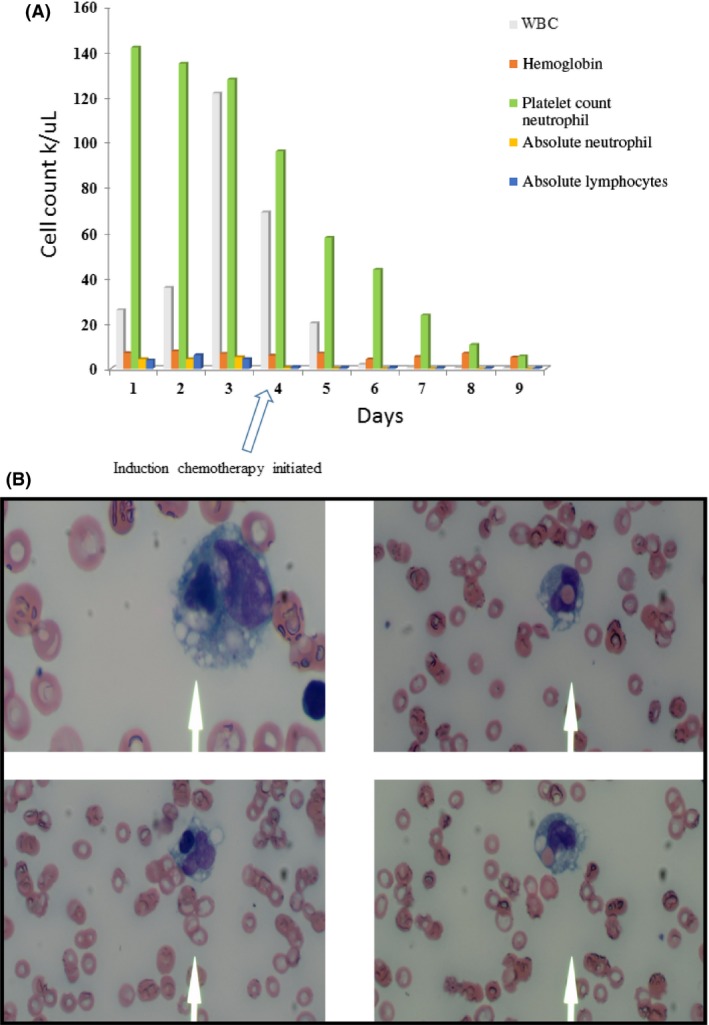
A, Laboratory tests representing actual blood counts peri‐induction. B, Peripheral blood smear with features of hemophagocytosis showing lymphocytes and red cells engulfed by macrophage (×400, Jenner Giemsa)

Studies have established that cytopenia following induction therapy for AML treatment is usually encountered from/around day ten post‐treatment. Hence, our question was whether this sudden cytopenia could be explained by any other process such as hemophagocytosis, bone marrow necrosis (BMN), or even extreme chemotherapy sensitivity of the leukemic cells.

A rare histopathological finding describing extensive bone marrow necrosis before or during treatment has been reported in AML patients 1, 2 resulting in varying degree of necrosis with ultimate result of cytopenia. Shapiro et al^1^ described a precipitous drop in blood pressure and tri‐lineage cell count following introduction of cytarabine, and reported bone marrow necrosis following autopsy.

Another entity that may describe the observed sudden pancytopenia in our patient is hemophagocytic lymphohistiocytosis (HLH). This represents the phagocytosis of erythrocytes, lymphocytes, or other hematopoietic precursors by histiocytes or macrophages either in bone marrow, lymph node, liver, or spleen. In fact, up to 10% of patients with AML undergoing chemotherapy have been reported to exhibit this entity, often associated with early mortality.^3^ This entity is usually accompanied by elevated ferritin level and triglyceride both of which were present in our patient thus making this pathology likely. Furthermore, the presence of soluble CD25 is seen with HLH.

Finally, one should not underestimate the chemo‐sensitivity of highly mitotic cells as encountered with acute leukemia. Although this is possible, it is quite unusual to occur so soon into therapy.

Unfortunately, our patient deteriorated few days following the severe cytopenia, requiring intubation and ICU management, and expired few days later from multiple organ failure. Attempts to obtain bone marrow biopsy proved abortive. We hypothesized that the observed clinical picture in our patient could be attributed to one of the three pathogenesis described above.

## CONFLICT OF INTEREST

None declared.

## AUTHORS CONTRIBUTION

DE, OL, RV, and BG: performed the chart review, collected all of the patient information, wrote the manuscript, and revised the manuscript. PK: edited the manuscript.
